# Synthesis and Sensing Applications of Fluorescent 3-Cinnamoyl Coumarins

**DOI:** 10.3390/s151229902

**Published:** 2015-12-19

**Authors:** Preeti Yadav, Hardeep Singh Gill, Karam Chand, Lian Li, Jayant Kumar, Sunil K. Sharma

**Affiliations:** 1 Department of Chemistry, University of Delhi, Delhi 110007, India; sandesh4preeti@gmail.com (P.Y.); kc4chemistry@gmail.com (K.C.); 2 Center for Advanced Materials, University of Massachusetts Lowell, Lowell, MA 01854, USA; hardeep_gill@student.uml.edu (H.S.G.); Lian_Li@uml.edu (L.L.); 3 Centro de Química Estrutural, Instituto Superior Técnico, Universidade de Lisboa, Av. Rovisco Pais 1, 1049-001 Lisboa, Portugal

**Keywords:** 3-(4-diethylaminocinnamoyl) coumarins, fluorescence, DCP, nerve gas sensors, two-photon fluorescence

## Abstract

We have synthesized two novel fluorescent 3-(4-diethylaminocinnamoyl) coumarins that exhibit fluorescence quenching upon exposure to a nerve agent simulant, diethylchlorophosphate (DCP), providing a basis for rapid and sensitive DCP chemosensing. Furthermore, these coumarin derivatives display two-photon fluorescence upon illumination with near-infrared laser pulses and their two-photon (TP) absorption cross-section was evaluated. The potential for TP bio-imaging of these compounds was investigated by their cellular uptake in HeLa cells by TP confocal microscopy.

## 1. Introduction

The extreme toxicity of organophosphorus (OP)-containing nerve agents such as Sarin, Soman, and Tabun poses a serious threat of chemical attack. Most of these nerve agents have chemical structures similar to those of insecticides and can irreversibly react with the enzyme acetylcholinesterase (a neurotransmitter), inhibiting its control over the central nervous system [1,2]. The use of such chemicals by terrorist groups in the past underscores the need to detect these odorless and colorless compounds. A variety of detection methods for nerve agents have been developed, including mass spectrometry-based techniques, enzyme-based sensors, colorimetric probes, and fluorometric methods [3,4,5,6,7,8,9,10]. However many of these systems suffer from their particular limitations, such as slow response times, operational complexity, and high cost, *etc.* Fluorescence-based detection methods are versatile due to their high sensitivity and much lower cost. Fluorescence-based sensors take advantage of the high electrophilicity of these nerve agents by reacting with a nucleophile embedded in a fluorophore leading to the change of fluorescence properties. The nerve agent simulants diethylcyanophosphate (DCNP), diethylchlorophosphate (DCP), and dimethylmethylphosphonate (DMMP) are normally used for research purposes as they offer similar reactivity as real nerve agents but lack the severe toxicity [11,12].

Coumarin-based derivatives have been widely used as fluorophores for constructing sensory systems for pH, metal cations, anions, and gases due to their desirable photophysical properties with large Stokes shifts and emissions in the visible spectral range [13,14,15,16,17,18,19]. Furthermore, these coumarin derivatives are also known to exhibit two-photon absorption (TPA) phenomena. Two-photon fluorescence (TPF)-based microscopy, a nonlinear optical microscopic technique, offers the advantages of increased penetration depth, localized excitation, and prolonged observation time, thus allowing tissue imaging [20,21,22,23]. However, the TPA cross-sections (δ) for the coumarin skeleton are too small to be useful in practical applications, as compared to those of other commercial dyes. Recent research suggests that the incorporation of a cinnamoyl moiety on the coumarin skeleton can lead to an enhancement of the TPA cross-section [24,25]. The TPA cross-sections for the 7-diethylamino-coumarin derivatives having the cinnamoyl moiety at C-3 were reported by Li *et al.* to be on the order of 60 to 360 GM and the cross-sections increased to 1349 and 1570 GM on further extending the conjugation at C-3 [24]. TPA cross-sections reported by Zou and coworkers for triethyleneglycol functionalized coumarin derivatives are on the order of 284 to 1556 GM [25].

Based on our interest in the design of chromofluorogenic probes for various photophysical and biological applications, we have synthesized a series of coumarin derivatives [26,27], of which 7-hydroxy-4-methylcoumarins bearing a 4-dimethylaminocinnamoyl moiety at the C-6/C-8 position have been explored earlier as molecular probes for confocal microscopy based bio-imaging [27]. In this study, we have incorporated a 4-diethylaminocinnamoyl moiety at the C-3 position of 7-methoxy/7,8-dimethoxy-4-hydroxycoumarin. An enhancement in the fluorescent intensity for the resulting compounds was observed, compared to the previously synthesized 4-dimethylaminocinnamoyl moiety at C-6/C-8 position of the coumarins [27]. These coumarin derivatives were used for sensing a nerve gas simulant, DCP, due to the presence of the two nucleophilic centers (NEt_2_ and OH). Upon treatment with DCP, both compounds exhibit significant fluorescence quenching. The Stern-Volmer constant and binding constant were calculated for these coumarins by fluorescence titration with DCP. Proton NMR spectra were recorded with the incremental amount of DCP in a solution of deuterated chloroform to identify the nucleophilic center involved in DCP sensing. In addition, these compounds were evaluated by the cellular uptake study of C-3 substituted cinnamoylcoumarins in HeLa cells by TPF microscopy for TP bio-imaging. The synthesized coumarin derivatives could be useful for nerve gas agent sensing and bio-imaging applications. 

## 2. Experimental Section 

### 2.1. Materials 

All of the chemicals and reagents were procured from Spectrochem Pvt. Ltd. (India) and Sigma-Aldrich (India). The organic solvents were dried and distilled prior to their use. Reactions were monitored by precoated TLC plates (silica gel 60F254, Merck, India); the spots were visualized either by UV light, or by spraying with 5% alcoholic FeCl_3_ solution. Silica gel (100–200 mesh) was used for column chromatography. 

### 2.2. Instruments 

Melting points were measured with a M-560 instrument (Buchi, Switzerland) and are uncorrected. Infrared spectra were recorded using a model 9 FT-IR spectrometer (Perkin-Elmer, Switzerland). ^1^H-NMR and ^13^C-NMR spectra (400 MHz and 100.5 MHz, respectively) were recorded employing a Jeol-400 spectrometer (Japan) using tetramethylsilane as the internal standard. The chemical shift values are on a δ scale and the coupling constant values (*J*) are in Hertz. The HRMS data were recorded on an Agilent-6530 Q-TOF LCMS (CA, USA). UV-Visible absorption spectra were recorded using a Cary Eclipse UV-Vis spectrophotometer (CA, USA). Photoluminescence spectra were recorded using a Fluorescence spectrophotometer (Cary 300) with both excitation and emission slit widths set at 2.5 nm (compounds **7**,**8**,**13**) and 5 nm (**11**,**12**). A quartz cuvette of 1 cm path-length was used to record the absorption and fluorescence spectra. 

### 2.3. Quantum Efficiency Measurement

For quantum efficiency measurements, the compounds were dissolved in DMSO (1 mg/mL) and diluted further to get the required concentration for optical studies. Commercially available rhodamine (RhB) dye was used as reference. Solutions with similar optical density were prepared. The relative quantum efficiency was measured using the following equation:

Ф_c_ = Ф_r_ × (F_c_/F_r_) × (n_c_/n_r_)^2^

where Ф_c_ = quantum efficiency of the 3-cinnamoyl coumarin, Ф_r_ = quantum efficiency of the reference, F_c_ = integrated fluorescence intensity of the 3-cinnamoyl coumarin, F_r_ = integrated fluorescence intensity of the reference, n_c_ = refractive index of the 3-cinnamoyl coumarin, n_r_ = refractive index of the reference. 

### 2.4. TPF Measurement

A mode-locked Ti:Sapphire laser (Quantronix, East Setauket, NY, USA) operated at 800 nm with a pulse width of 100 femto-second and a repetition rate of 1 kHz was used as the excitation source for the TPF study. The near-infrared (NIR) laser pulses were passed through the sample cuvette and the fluorescence signal was collected at 90° with respect to the laser beam using a CCD and a monochromator. A short-pass optical filter at 750 nm was placed in front of the monochromator to eliminate the excitation radiation. A short focal length (4.5 cm) lens was employed to collect the TPF signal.

### 2.5. TPA Cross Section Measurement

The TPA cross-sections were determined using RhB in DMSO as a reference. The TPA cross-sections were calculated using the following formula:

δ_c_ = δ_r_(F_c_/F_r_)(Ф_r_/Ф_c_)(C_r_/C_c_)

where Fs are the integrated TPF intensities; Cs are the concentrations; subscript c and r stand for the 3-cinnamoyl coumarin and the reference molecule, respectively [28].

### 2.6. Stern-Volmer Constant (Ksv), Binding Constant (K) and Number of Binding Sites (n) Measurements

The Stern-Volmer constant was calculated from the fluorescence titration data of compounds **7** or **8** (0.02 mM, 3 mL), with increasing concentration of DCP in chloroform and by plotting the relative fluorescence intensity (I_o_/I) against the [DCP] (Figure S13) using the following equation:

I_o_/I = 1 + K_sv_ × [DCP]

The plot between log[(I_o_ − I)/I] and log[DCP], gives rise to the binding constant (K) and number of binding sites (n) in compound **7** or **8** for DCP (Figure S14) was determined using the equation below:

log[(I_o_ − I)/I] = logK + n × log[DCP]

where I_o_ and I correspond to emission intensities at 573 nm (**7**) or 572 nm (**8**) in the absence and presence of DCP, respectively. For the absorption spectra measurements, 0.85 mL solution of compounds **7** or **8** (0.02 mM) was used.

### 2.7. Chemistry

#### 2.7.1. 3-Acetyl-4-hydroxy-7,8-dimethoxy-2*H*-chromen-2-one (**6**)

Compound **6** was obtained as an off white solid in 88% yield from the reaction of 4-hydroxy-7,8-dimethoxy-2*H*-chromen-2-one (4) with acetic acid and phosphorus oxychloride. Melting point: 163-164 °C; IR (KBr) ν_max_: 2926.40, 1726.24 (OCO), 1604.25 (CO), 1553.39, 1476.12, 1297.03, 1098.71, 1048.65, 993.50, 758.46, 667.85 cm^−1^; ^1^H-NMR (CDCl_3_): δ 2.75 (s, 3H, COCH_3_), 3.97 (d, 3H, *J* = 1.52 Hz, 7-OCH_3_), 4.00 (s, 3H, 8-OCH_3_), 6.93 (d, 1H, *J* = 9.16 Hz, H-5), 7.79 (dd, 1H, *J* = 1.52, 9.16 Hz, H-6); ^13^C-NMR (CDCl_3_): δ 29.96, 56.51, 61.57, 99.93, 108.62, 109.42, 121.43, 135.73, 148.80, 158.90, 159.82, 178.65, 205.64; HRMS: Calculated for C_13_H_13_O_6_ [M+H]^+^ 265.0712, found 265.0717.

#### 2.7.2. (*E*)-3-[3-(4-(Diethylaminophenyl)acryloyl]-4-hydroxy-7-methoxy-2*H*-chromen-2-one (**7**)

Compound **7** was obtained as a red solid in 78% yield from the reaction of 3-acetyl-4-hydroxy-7-methoxy-2*H*-chromen-2-one (**5**) with 4-diethylaminobenzaldehyde. Melting point: 232-233 °C; IR (KBr) ν_max_: 2926.52, 1713.43 (OCO), 1634.33 (CO), 1592.85, 1521.42, 1457.13, 1288.37, 1194.09, 1098.57, 955.49, 693.02 cm^−1^; ^1^H-NMR (CDCl_3_): δ 1.22 (t, 6H, *J* = 6.48 Hz, 2 × NCH_2_*CH_3_*), 3.44 (q, 4H, *J* = 6.88 Hz, 2 × N*CH_2_*CH_3_), 3.89 (s, 3H, OCH_3_), 6.66 (d, 2H, *J* = 8.4 Hz, H-3′′ & H-5′′), 6.72 (d, 1H, *J* = 2.32 Hz, H-8), 6.86 (dd, 1H, *J* = 2.28, 8.4 Hz, H-6), 7.62 (d, 2H, *J* = 9.16 Hz, H-2′′ & H-6′′), 7.97 (d, 1H, *J* = 8.4 Hz, H-5), 8.07 (d, 1H, *J* = 15.28 Hz, H-2′), 8.20 (d, 1H, *J* = 15.24 Hz, H-3′); ^13^C-NMR (CDCl_3_): δ 12.60, 44.64, 55.88, 98.60, 100.07, 110.67, 111.30, 112.70, 115.22, 122.27, 127.15, 132.39, 148.88, 150.75, 156.62, 160.92, 165.67, 181.82, 190.13; HRMS: Calculated for C_23_H_23_NO_5_ [M+H]^+^ 394.1654, found 394.1672 and [M+Na]^+^ 416.1474, found 416.1476.

#### 2.7.3. (*E*)-3-[3-(4-(Diethylaminophenyl)acryloyl]-4-hydroxy-7,8-dimethoxy-2*H*-chromen-2-one (8)

Compound **8** was obtained as a red solid in 72% yield from the reaction of 3-acetyl-4-hydroxy-7,8-dimethoxy-2*H*-chromen-2-one (**6**) with 4-diethylaminobenzaldehyde. Melting point: 193-194 °C; IR (KBr) ν_max_ : 2922.26, 1712.17 (OCO), 1582.71 (CO), 1520.13, 1438.03, 1094.38, 814.60 cm^−1^; ^1^H- NMR (CDCl_3_): δ 1.22 (t, 6H, *J* = 6.88 Hz, 2 × NCH_2_*CH_3_*), 3.44 (q, 4H, *J* = 6.88 Hz, 2 × N*CH_2_*CH_3_), 3.98, 3.99 (2s, 6H, 2 × OCH_3_), 6.66 (d, 2H, *J* = 7.64 Hz, H-3′′ & H-5′′), 6.89 (d, 1H, *J* = 9.16 Hz, H-5), 7.61 (d, 2H, *J* = 8.4 Hz, H-2′′ & H-6′′), 7.82 (d, 1H, *J* = 9.16 Hz, H-6), 8.08 (d, 1H, *J* = 15.28 Hz, H-2′), 8.21 (d, 1H, *J* = 15.28 Hz, H-3′); ^13^C-NMR (CDCl_3_): δ 12.35, 44.74, 56.41, 61.52, 98.58, 108.21, 111.61, 121.40, 132.24, 135.60, 148.53, 158.33, 160.26, 181.82, 190.04; HRMS: Calculated for C_24_H_25_NO_6_ [M+H]^+^ 424.1760, found 424.1770 and [M+Na]^+^ 446.1580, found 446.1587.

#### 2.7.4. (*E*)-6-[3-(4-Diethylaminophenyl)acryloyl]-7-hydroxy-4-methyl-2*H*-chromen-2-one (12)

Compound **12** was obtained as a red solid in 78% yield from the reaction of 6-acetyl-7-hydroxy-2*H*-chromen-2-one (**9**) with 4-diethylaminobenzaldehyde. Melting point: 244-245 °C; IR (KBr) ν_max_: 3434.64 (OH), 2926.45, 1716.98 (OCO), 1632.08 (CO), 1521.64, 1303.88, 1182.45, 1151.44, 1088.49, 884.61 cm^−1^; ^1^H-NMR (CDCl_3_): δ 1.15 (t, 6H, *J* = 6.88 Hz, 2 × NCH_2_*CH_3_*), 2.40 (s, 3H, C-4 CH_3_), 3.41 (q, 4H, *J* = 6.88 Hz, 2 × N*CH_2_*CH_3_), 6.09 (s, 1H, H-3), 6.60 (d, 2H, *J* = 9.16 Hz, H-3′′ & H-5′′), 6.78 (s, 1H, H-8), 7.26 (d, 1H, *J* = 14.52 Hz, H-2′), 7.49 (d, 2H, *J* = 9.16 Hz, H-2′′ & H-6′′), 7.88 (d, 1H, *J* = 15.24 Hz, H-3′), 8.02 (s, 1H, H-5), 13.75 (s, 1H, OH); ^13^C-NMR (CDCl_3_): δ 12.55, 18.67, 44.63, 105.29, 111.28, 112.25, 112.47, 117.80, 121.11, 126.42, 131.56, 147.88, 150.43, 152.00, 158.15, 160.29, 166.55, 192.06; HRMS: Calculated for C_23_H_23_NO_4_ [M+H]^+^ 378.1705, found 378.1706.

#### 2.7.5. (*E*)-6-[3-(4-Diethylaminophenyl)acryloyl]-7-methoxy-4-methyl-2*H*-chromen-2-one (13)

Compound **13** was obtained as a yellow solid in 90% yield from the reaction of (*E*)-6-[3-(4-diethyl-aminophenyl)acryloyl]-7-hydroxy-4-methyl-2*H*-chromen-2-one (**12**) with methyl iodide. Melting point: 208–209 °C; IR (KBr) ν_max_: 2926.64, 1720.49 (OCO), 1609.44 (CO), 1559.14, 1363.70, 1278.58, 1160.45, 1083.93, 984.66, 812.14 cm^−1^; ^1^H-NMR (CDCl_3_): δ 1.20 (t, 6H, *J* = 6.88 Hz, 2 × NCH_2_*CH_3_*), 2.42 (s, 3H, C-4 CH_3_), 3.41 (q, 4H, *J* = 6.88 Hz, 2 × N*CH_2_*CH_3_), 3.95 (s, 3H, OCH_3_), 6.17 (s, 1H, H-3), 6.64 (d, 2H, *J* = 9.16 Hz, H-3′′ & H-5′′), 6.88 (s, 1H, H-8), 7.13 (d, 1H, *J* = 15.24 Hz, H-2′), 7.46 (d, 2H, *J* = 9.16 Hz, H-2′′ & H-6′′), 7.59 (d, 1H, *J* = 16.04 Hz, H-3′), 7.85 (s, 1H, H-5); ^13^C-NMR (CDCl_3_): δ 12.54, 18.71, 44.48, 56.24, 99.64, 111.21, 112.49, 113.30, 120.77, 121.55, 127.02, 127.10, 130.84, 145.66, 149.73, 152.79, 156.58, 160.69, 191.04; HRMS: Calculated for C_24_H_25_NO_4_ [M+H]^+^ 392.1862, found 392.1861.

## 3. Results and Discussion

### 3.1. Synthesis

Compounds 4-hydroxy-7-methoxy-2*H*-chromen-2-one (**3**) and 4-hydroxy-7,8-methoxy-2*H*-chromen-2-one (**4**) were synthesized by the reaction of 4-methoxy-2-hydroxyacetophenone (**1**) and 3,4-dimethoxy-2-hydroxyacetophenone (**2**), respectively, with diethyl carbonate in the presence of sodium hydride [29], and were then subjected to acetylation with acetic acid and phosphorus oxychloride to yield respective acetylated compounds **5** and **6** [30].

**Scheme 1:**
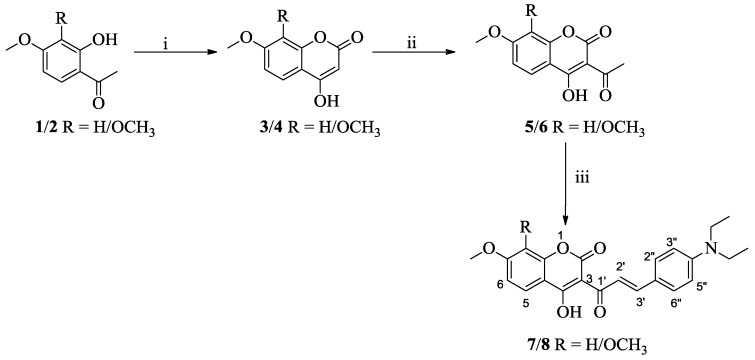
Synthesis of compounds **5**–**8**. *Reagents and conditions*: i. NaH, diethyl carbonate; ii. acetic acid, phosphorus oxychloride, reflux; iii. 4-diethylaminobenzaldehyde, two drops of piperidine, ethanol, reflux.

3-(4-Diethylaminocinnamoyl) coumarins **7**,**8** were synthesized by piperidine catalyzed Claisen-Schmidt condensation of 4-diethylaminobenzaldehyde with 3-acetyl-4-hydroxy-7-methoxy-2*H*-chromen-2-one (**5**) or 3-acetyl-4-hydroxy-7,8-dimethoxy-2*H*-chromen-2-one (**6**) (Scheme 1). The compounds **6**–**8** are novel and were synthesized for the first time. They were fully characterized by ^1^H-, ^13^C-NMR and mass spectroscopy (see Figures S1–S6 in the Supporting Information (SI)). 

**Scheme 2:**
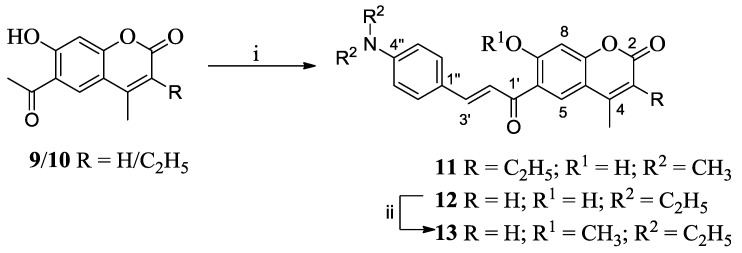
Synthesis of compounds **11**–**13**. *Reagents and conditions*: i. 4-dialkylamino-benzaldehyde, two drops of piperidine, ethanol, reflux; ii. CH_3_I, dimethylformamide, K_2_CO_3_, 30 °C.

The C-6 substituted 4-dialkylaminocinnamoylcoumarins 11 and 12 were synthesized by Claisen-Schmidt condensation of 4-dialkylaminobenzaldehyde with 6-acetyl-7-hydroxy-3-alkyl-4-methyl coumarins 9, 10. Compound **13** was synthesized by carrying *O*-methylation of compound **12** using methyl iodide (Scheme 2). The compounds **12** and **13** are novel and synthesized for the first time. They were fully characterized by ^1^H, ^13^C-NMR and mass spectroscopy (Figures S7–S10).

### 3.2. One-Photon and TP Activity of 3-Cinnamoylcoumarins

UV-Vis absorption and fluorescence spectra of 3-(4-diethylaminocinnamoyl) coumarins 7 and 8 were recorded by dissolving the compounds in DMSO. These compounds exhibit strong absorption maxima at 521 and 523 nm, respectively. Upon exciting at their absorption maxima range, they emit strong fluorescence with λ_max_ at 608 and 610 nm, respectively (Figure S11). Stokes shift of 87 nm was observed for these compounds. The solvatochromism effect of these compounds was also studied and shown in Figure S12, Table S1. The quantum yields (Ф) of the compounds were measured using RhB as a reference in DMSO having quantum efficiency of 96% [31]. The quantum yields of these compounds were determined and summarized in Table 1.

**Table 1:** Optical properties of the 3-(4-diethylaminocinnamoyl) coumarins in DMSO.

Compd.	λ_Ab._ (nm)	λ_Em._ (nm)	Stokes Shift (nm)	Ф (%)	Δ (GM)
7	521	608	87	11.01	59
8	523	610	87	17.60	85

**Figure 1:**
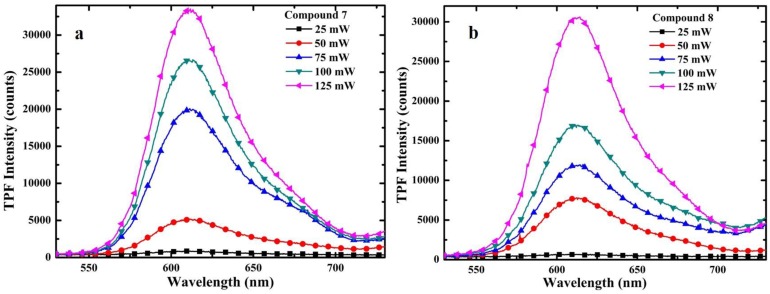
TPF spectra (**a**) for compound **7** and (**b**) for compound **8**.

**Figure 2:**
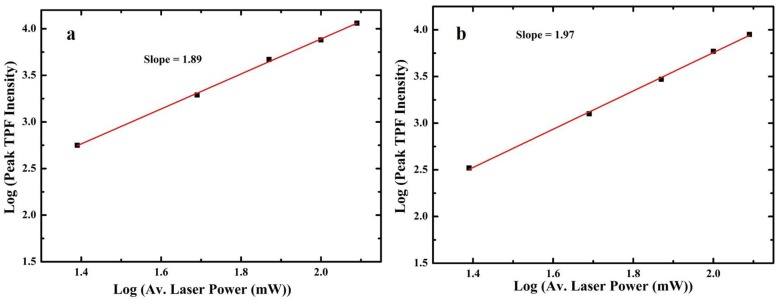
Quadratic dependence of TPF (**a**) for compound **7** and (**b**) for compound **8**.

The TPF spectra of these compounds were recorded by exciting them with the NIR femtosecond laser pulses in dilute DMSO solution. Figure 1 shows the intensity dependent TPF spectra for compounds **7** and **8**. In both of the cases, an increase in the TPF intensity with the increase of the exciting laser power was observed. The quadratic behavior of the TPF was confirmed by plotting log[peak TPF intensity] *vs* log[laser power] as shown in Figure 2. A slope close to two in the plot was determined, indicating that the fluorescence from the 3-(4-diethylaminocinnamoyl) coumarins 7 or 8 is indeed a TP phenomenon. The TPA cross-sections of 7 and 8 were then calculated, using the RhB as a reference having TPA cross-section of 28 GM at 800 nm [32]. The TPA cross-sections of the compounds studied (Table 1) were found to be comparable to the dyes used as biomarkers [33]. 

A correlation of quantum efficiency and TPA cross-sections with the chemical structures of the coumarins was observed. An enhancement in quantum efficiency and TPA cross-section was observed on substituting the C-8 position of 3-(4-diethylaminocinnamoyl) coumarins with a methoxy group. A similar effect of electron donating group on δ value of same conjugated system was observed byLi *et al.* [24]. Higher TPA cross-section and quantum yield for coumarin derivative 8 were measured as compared to those for coumarin derivative **7**. This observation could be helpful for the future design and development of the coumarin based TP fluorophores.

### 3.3. TPF Imaging 

**Figure 3:**
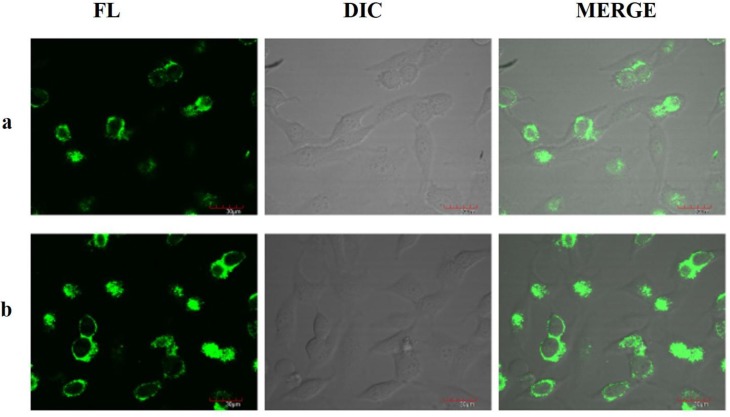
TP confocal images of HeLa cells (**a**) with compound **7** and (**b**) compound **8**. FL: coumarin fluorescence images; DIC: (differential interference contrast images); and MERGE: Fluorescence images merged with DIC.

TPF microscopy confirmed the cellular uptake of these 3-cinnamoylcoumarins in HeLa cells, revealing the potentials of these coumarins as TP probes. For compounds **7** and **8**, TP confocal imaging was carried out using a 710 Laser Scanning Microscope (Zeiss, Jena, Germany). HeLa cells were grown in DMEM containing 10% fetal bovine serum at 37 °C. Cinnamoyl -coumarins (2 μg/mL) were incubated for 30 minutes in dish (P35G-1.5-10-C, MatTek Corporation, Ashland, MA, USA), and the uptake was directly analyzed under the microscope. Figure 3 shows the TPF confocal images of the uptake of the 3-cinnamoylcoumarin molecules in HeLa cells. The cellular uptake results suggest that the synthesized coumarin compounds could be used as potential fluorescent probes for TP imaging.

### 3.4. DCP Sensing Study

**Figure 4:**
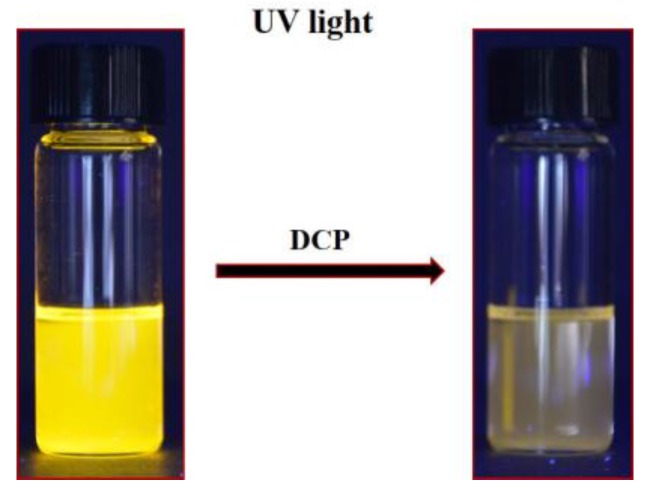
Color change observed on addition of DCP (4 eq.) to a solution of compound **8** in CHCl_3_.

**Figure 5:**
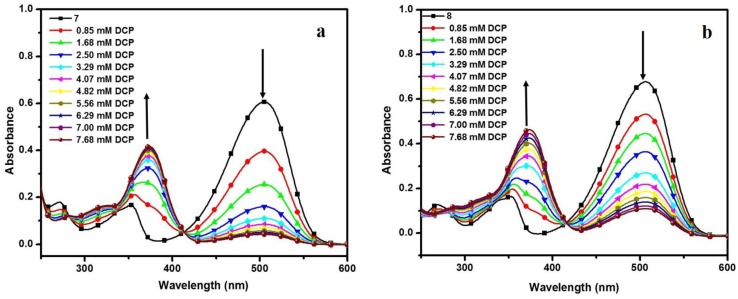
UV-Vis absorption spectra upon titration of compounds 7 (**a**) and 8 (**b**) with DCP.

To study the DCP sensing capability of 3-cinnamoylcoumarins 7 and 8, the absorption and the fluorescence spectra of the compounds was recorded in chloroform with the addition of different concentrations of DCP. The solutions of compounds **7** and **8** in chloroform absorb strongly around 506 and 505 nm, and emit bright orange fluorescence with λ_max_ at 573 and 572 nm upon excitation, respectively. The fluorescence quenching of both compounds **7** and **8** with DCP in the chloroform solutions were observed (Figure 4). Both solutions turned colorless upon addition of DCP. The absorption peaks at 506 and 505 nm for compounds **7** and **8** kept decreasing with the appearance of a new absorption band at 372 nm. Figure 5 presents the absorption spectra of compounds **7** and **8** upon titrating with DCP (0.85–7.68 mM). A blue shift in the absorption maxima of compounds **7** and **8** was determined to be about 133 nm on the incremental addition of DCP. This change in UV absorption that accompanies DCP binding *i.e.*, one peak goes down and the other goes up may be useful for ratiometric measurements.

**Figure 6:**
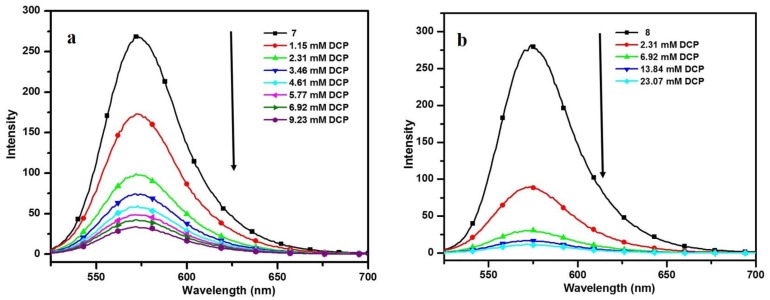
Fluorescence spectra upon titration of compounds **7** (**a**) and 8 (**b**) with DCP.

Upon addition of DCP to chloroform solution of cinnamoylcoumarins 7 and 8, the fluorescence intensities at 573 and 572 nm decreased as shown in Figure 6. The corresponding Stern-Volmer plots are shown in Figure S13 and the Stern-Volmer constant (Ksv), the binding constant (K) and number of binding sites (n) (Figure S14) were calculated and are listed in Table 2. It has been observed that the substitution of the methoxy group at the C-8 position of the coumarin increases the value of the Stern-Volmer constant and the binding constant. This may be due to the electron donating effect of methoxy group which enriched the nucleophilic attack of compound on phosphorous atom of DCP. The Ksv values for both compounds **7** and **8** were found to be higher than the value reported for the DCP sensor based on poly(fluorene-quinoxaline) [34]. To confirm if the fluorescence quenching is a selective phenomenon for the cinnamoylcoumarins **7** and **8**, we have synthesized three structural analogues (11−13) of compounds **7** and **8** (Scheme 2). The TPA activity of compound **11** was previously reported by our group, but the DCP sensing application has not been explored earlier [27]. For these analogues (11−13), although a similar tendency of fluorescence quenching has been observed on incremental addition of DCP (Figures S15–S17 in SI) as for the compounds **7** and **8**. However, the measured Stern-Volmer constants revealed that these analogues of 7 and 8 exhibit weaker quenching responses with DCP as described in Table S2. The binding constants for the synthesized compounds are comparable with the value reported for rhodamine-based sensors for DCP [35].

**Table 2:** The Stern-Volmer constants, binding constants, and number of binding sites of the 3-(4-diethylaminocinnamoyl)coumarins with DCP in chloroform.

Compd.	λ_Ab._ (nm)	λ_Em._ (nm)	Ksv (M^−1^)	K (M^−1^)	n
**7**	506	573	791	8.235 X 10^2^	1.011
**8**	505	572	1070	1.410 X 10^3^	1.054

In order to evaluate the selectivity of compounds **7** and **8** for different organophosphorous compounds, we studied the change in the fluorescence intensity of compound **7** towards two more organophosphorous compounds, *i.e.*, orthophosphoric acid and dimethyl methylphosphonate. Quite interestingly, no significant change in the emission spectra of compound **7** was observed with the addition of any of the two compounds (Figure S18) even with addition of 30 times higher amount of orthophosphoric acid/dimethyl methylphosphonate in comparison to DCP (9.23 mM). This result clearly showed that compounds **7** and **8** are highly selective towards sensing the DCP. 

### 3.5. NMR Study and the Sensing Mechanism

**Figure 7:**
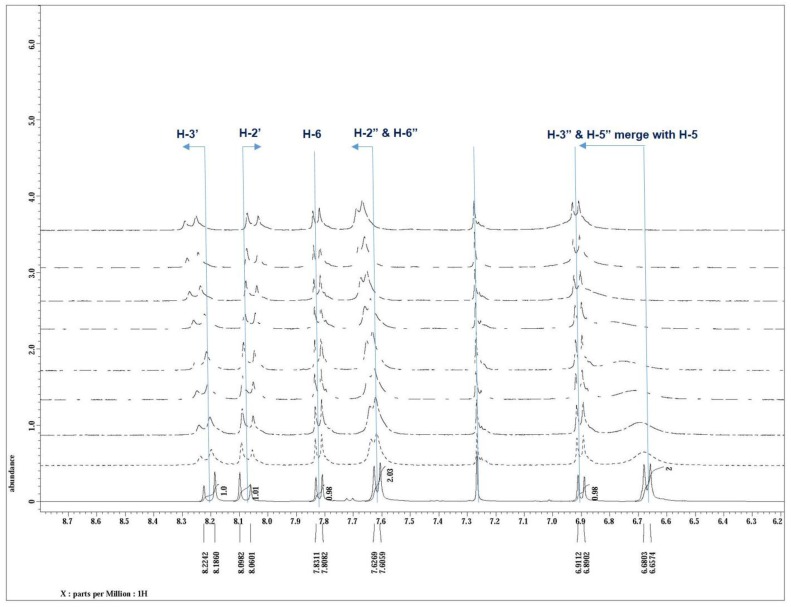
^1^H-NMR spectra of compound **8** in CDCl_3_ showed shifts in aromatic protons on the addition of DCP (0–4 eq.).

A ^1^H-NMR titration study was performed to find the nucleophilic center (NEt_2_ or OH) involved in the binding of DCP. Interestingly, it was noticed that the continuous addition of DCP (0–4 eq.) to compound **8** resulted in a deshielding of the protons *ortho* (H-3′′ & H-5′′) to the NEt_2_ group (Figure 7). A downfield shift in the peak signal of the methylene protons [N(CH_2_CH_3_)_2_] as well as *meta*-protons (H-2′′ & H-6′′) was observed on addition of DCP (Figure S19), while the benzenoid protons of the coumarin skeleton ring (H-5 & H-6) did not exhibit any significant change in δ value, this may be due to the safe distance from the reactive nucleophilic center involved with DCP interaction. These facts suggest that the participation of NEt_2_ group in nucleophilic attack on phosphorous atom of DCP. 

**Scheme 3:**
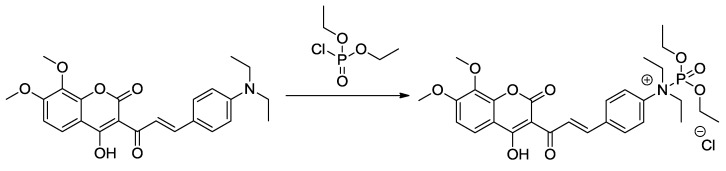
Interaction of DCP with compound **8**.

This downfield shift of H-3′′ & H-5′′ on DCP addition is attributed to change from the +R to –R effect of NEt_2_ group after reacting with DCP. The protonation of nitrogen leads to withdraw of electron density from the 3′ proton, resulting in the increase in δ value of this proton and upfield shift of 2′ proton. A similar effect on chemical shift values was observed in the ^1^H-NMR of compound **7** on DCP addition (Figure S20), the proton NMR of compound **7** was recorded on addition of DCP (4 equivalent) at the time interval of 24 and 48 h (Figure S21). Even after 48 h, no significant shift was observed in the protons of coumarin moiety (H-5, H-6, and H-8). Based on this observation, a possible mechanism of DCP binding that triggers the quenching of the fluorescence intensity of compound **8** was postulated and shown in Scheme 3. 

## 4. Conclusions

Two novel 3-(4-diethylaminocinnamoyl)coumarins 7,8 were synthesized by Claisen-Schmidt condensation of 4-diethylaminobenzaldehyde with 3-acetyl-4-hydroxy-7-methoxy-2*H*-chromen-2-one or 3-acetyl-4-hydroxy-7,8-dimethoxy-2*H*-chromen-2-one. These coumarins exhibited significant Stokes shifts, moderate fluorescence quantum efficiencies and TPA cross-sections. A correlation of the chemical structure and TPF was established. The TPF confocal microscopic investigation confirmed the internalization of these 3-cinnamoyl coumarins by HeLa cell lines. These compounds showed large fluorescence quenching upon addition of DCP. The Ksv values obtained for the compounds **7** and **8** by the fluorescence titration method were found to be 791 and 1070 M^−1^, respectively. These values are comparable or even higher than those reported for other nerve gas sensors. Thus, we have successfully developed a reagent that exhibit good selectivity to interact with DCP in comparison to its structural analogues DMMP and orthophosphoric acid. Our research findings could be useful for future design of coumarin based biological imaging probes and nerve gas sensors.

## Supplementary Files

Supplementary File 1
