# Supplementary material for: Synthesis and Sensing Applications of Fluorescent 3-Cinnamoyl Coumarins
Source: Sensors (Basel). 2015 Dec 19;15(12):31987–98. doi: 10.3390/s151229902 (PMC4721820; doi:10.3390/s151229902)
Supplement: Supplementary File 1 [file sensors-15-29902-s001.pdf]

# Supplementary Materials: Synthesis and Sensing Applications of Fluorescent 3-Cinnamoyl Coumarins

**Preeti Yadav, Hardeep Singh Gill, Karam Chand, Lian Li, Jayant Kumar and Sunil K. Sharma**

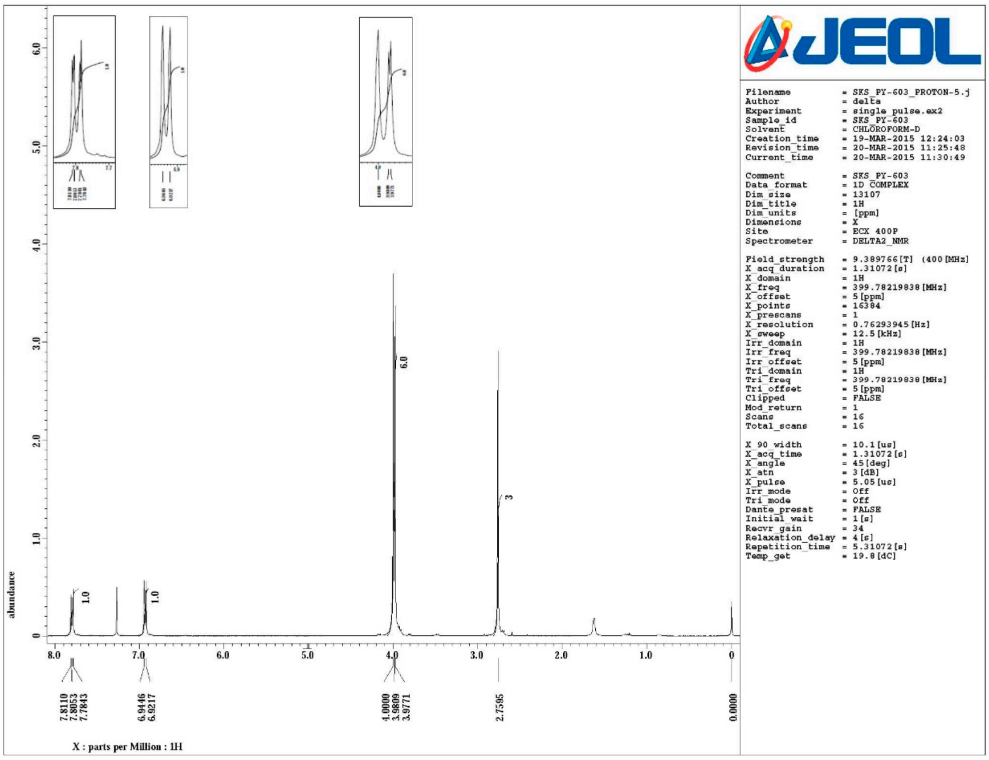


**Figure S1.** 1H-NMR of 3-acetyl-4-hydroxy-7,8-dimethoxy-2H-chromen-2-one (**6**).

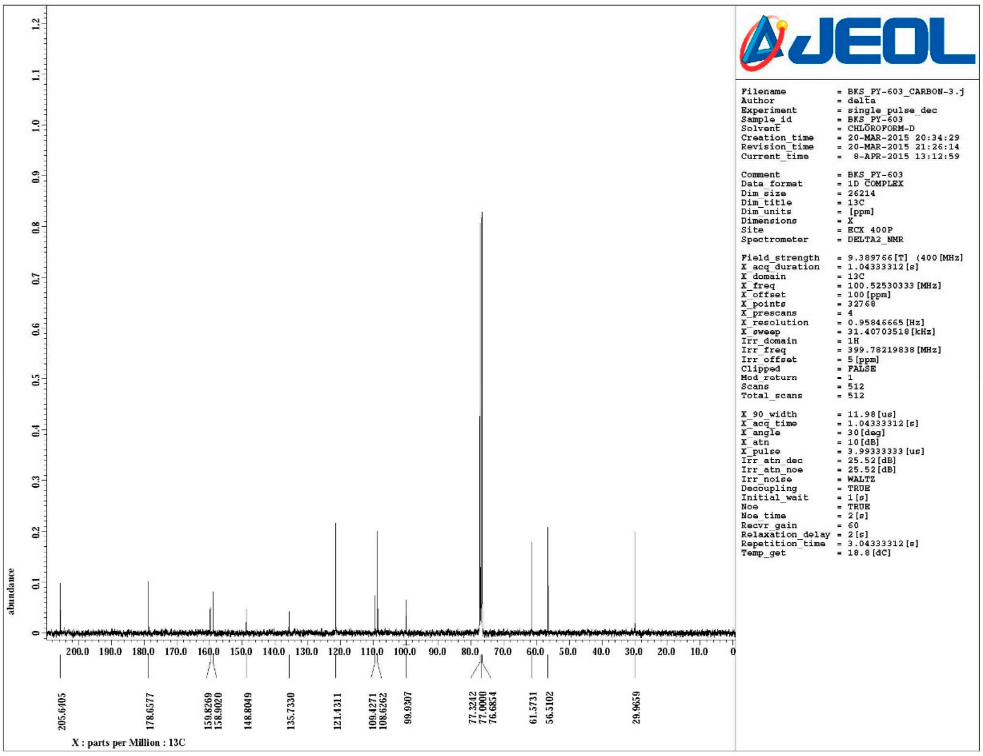


**Figure S2.** 13C-NMR of 3-acetyl-4-hydroxy-7,8-dimethoxy-2H-chromen-2-one (**6**).

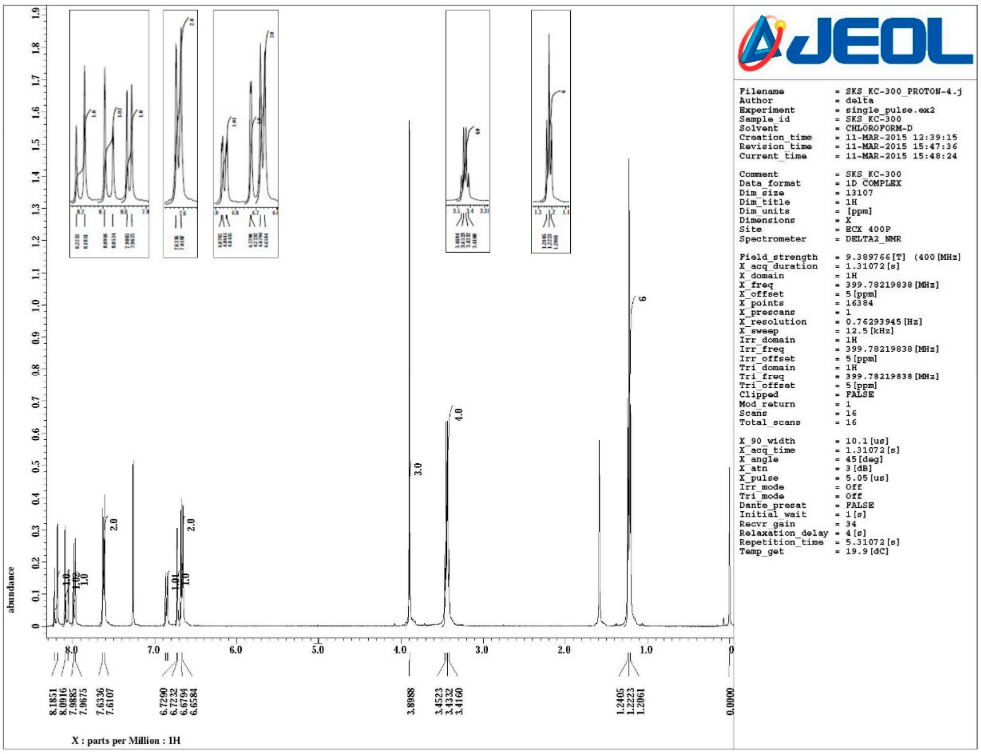


**Figure S3.** 1H-NMR of (E)-3-[3-(4-(diethylaminophenyl)acryloyl]-4-hydroxy-7-methoxy-2H-chromen-2-one (**7**).

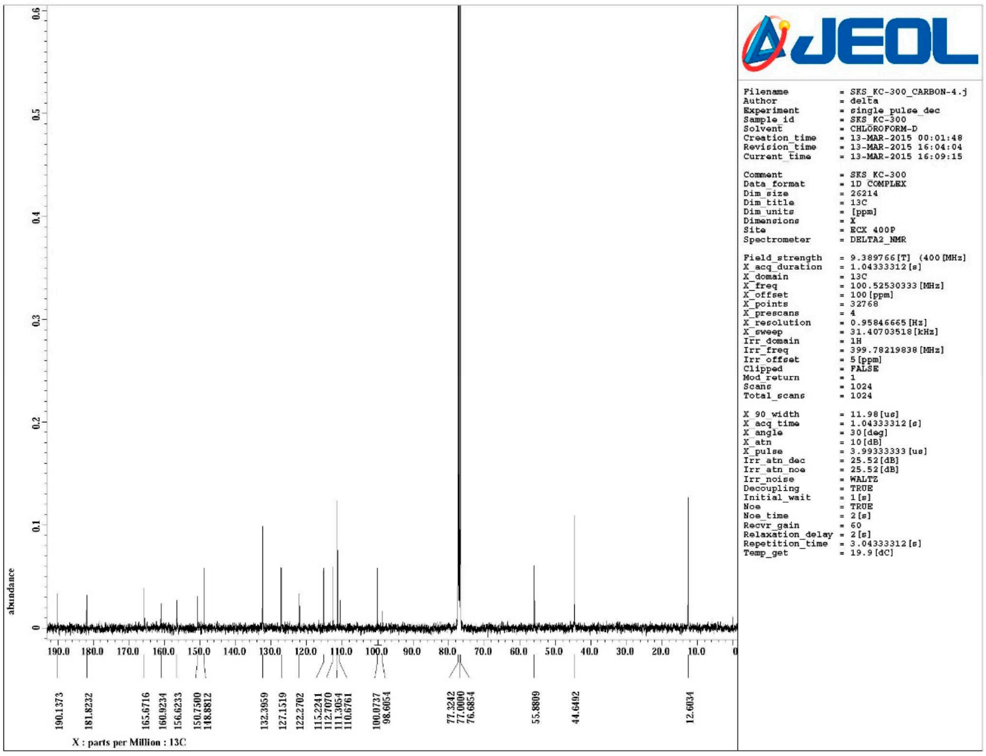


**Figure S4.** 13C-NMR of (E)-3-[3-(4-(diethylaminophenyl)acryloyl]-4-hydroxy-7-methoxy-2H-chromen-2-one (**7**).

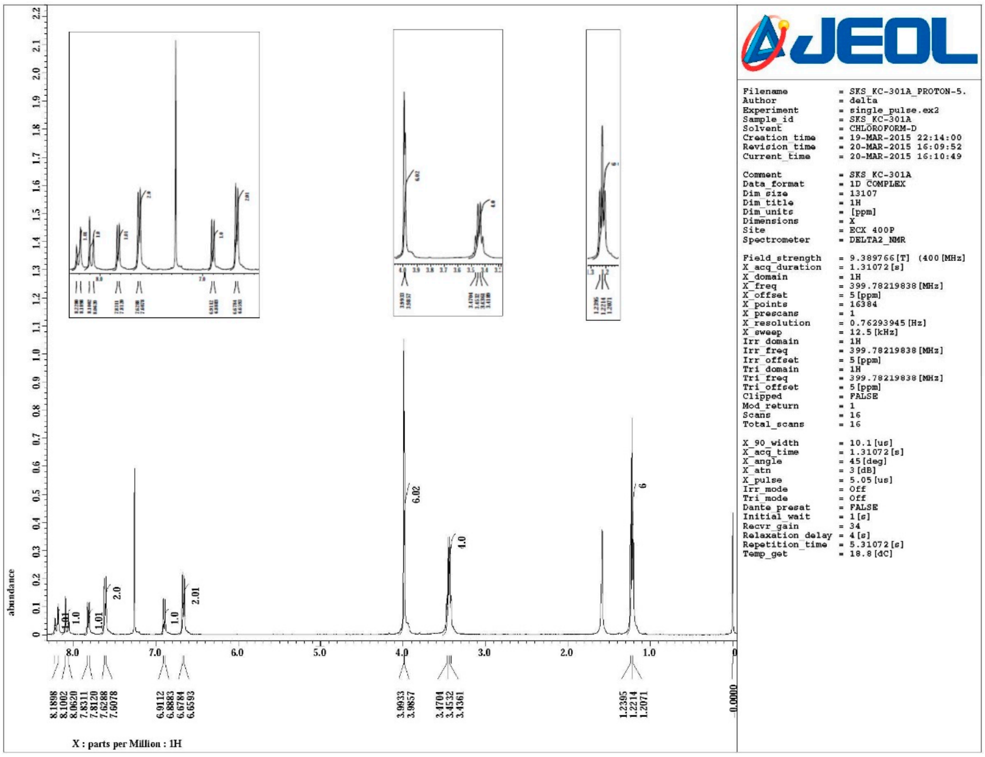


**Figure S5**. 1H-NMR of (E)-3-[3-(4-(diethylaminophenyl)acryloyl]-4-hydroxy-7,8-dimethoxy-2H-chromen-2-one (**8**).

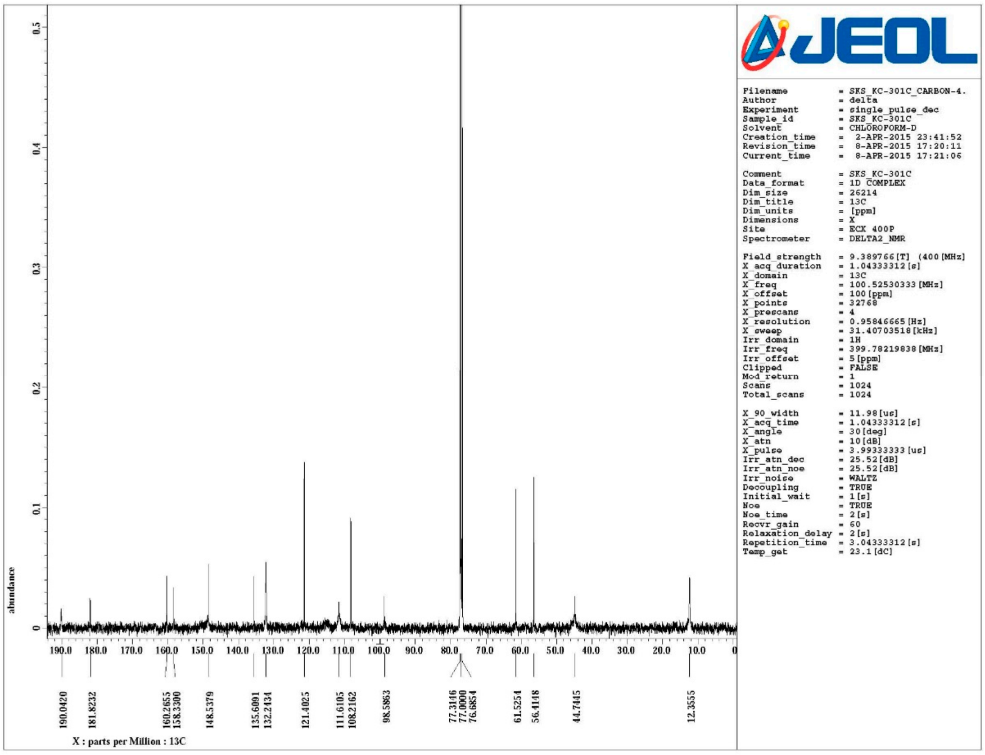


**Figure S6.** 13C-NMR of (E)-3-[3-(4-(diethylaminophenyl)acryloyl]-4-hydroxy-7,8-dimethoxy-2H-chromen-2-one (**8**).

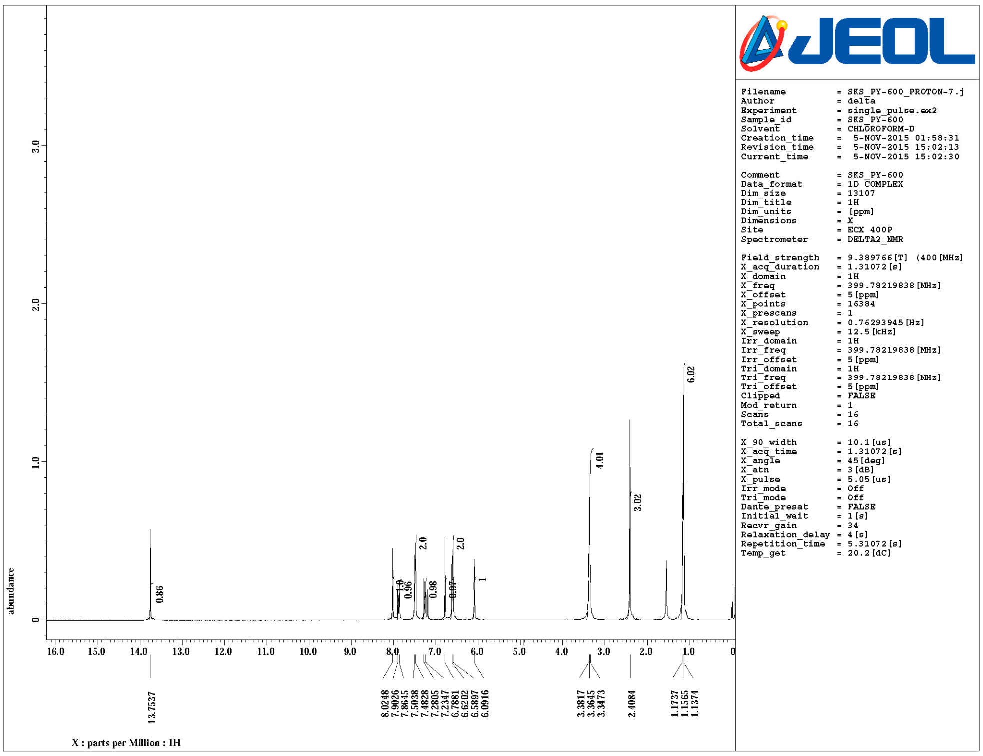


**Figure S7.** 1H-NMR of (E)-6[(3-(4-diethylaminophenyl)acryloyl]-7-hydroxy-4-methyl-2H-chromen-2-one (**12**).

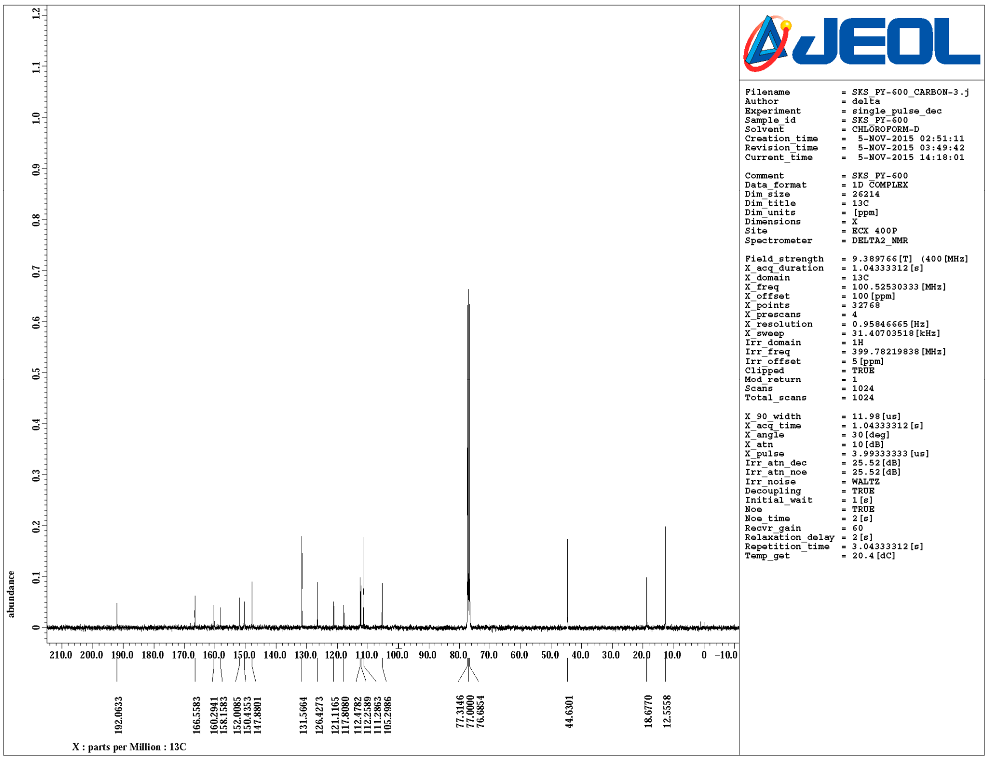


**Figure S8.** 13C-NMR of (E)-6[(3-(4-diethylaminophenyl)acryloyl]-7-hydroxy-4-methyl-2H-chromen-2-one (**12**).

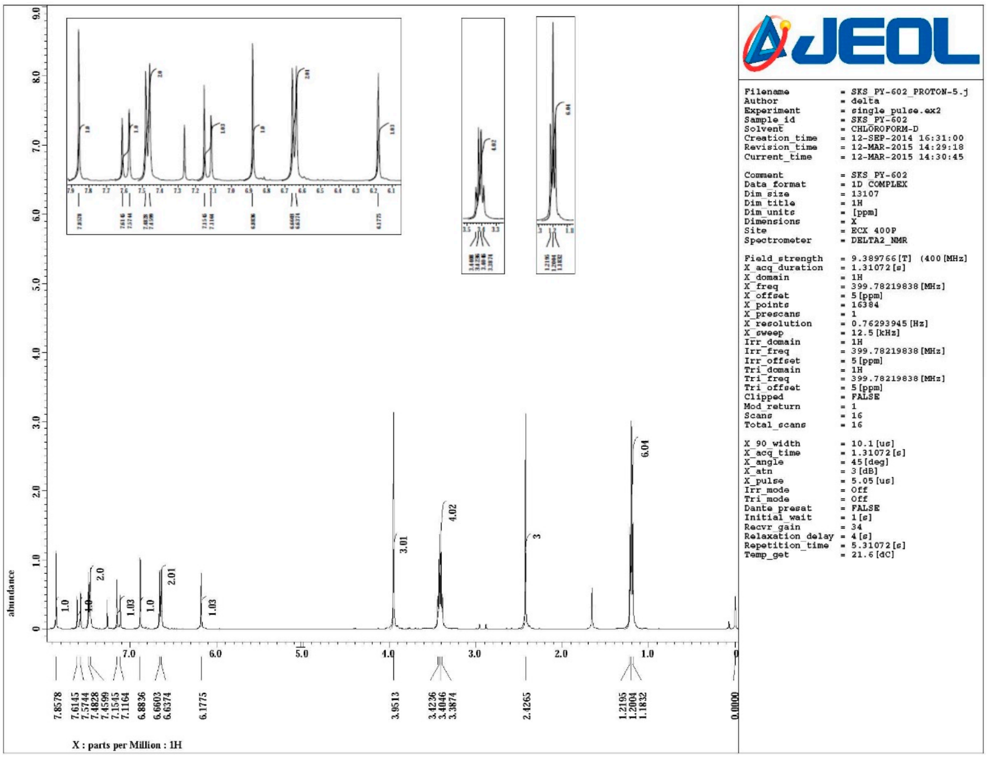


**Figure S9.** 1H-NMR of (E)-6[(3-(4-diethylaminophenyl)acryloyl]-7-methoxy-4-methyl-2H-chromen-2-one (**13**).

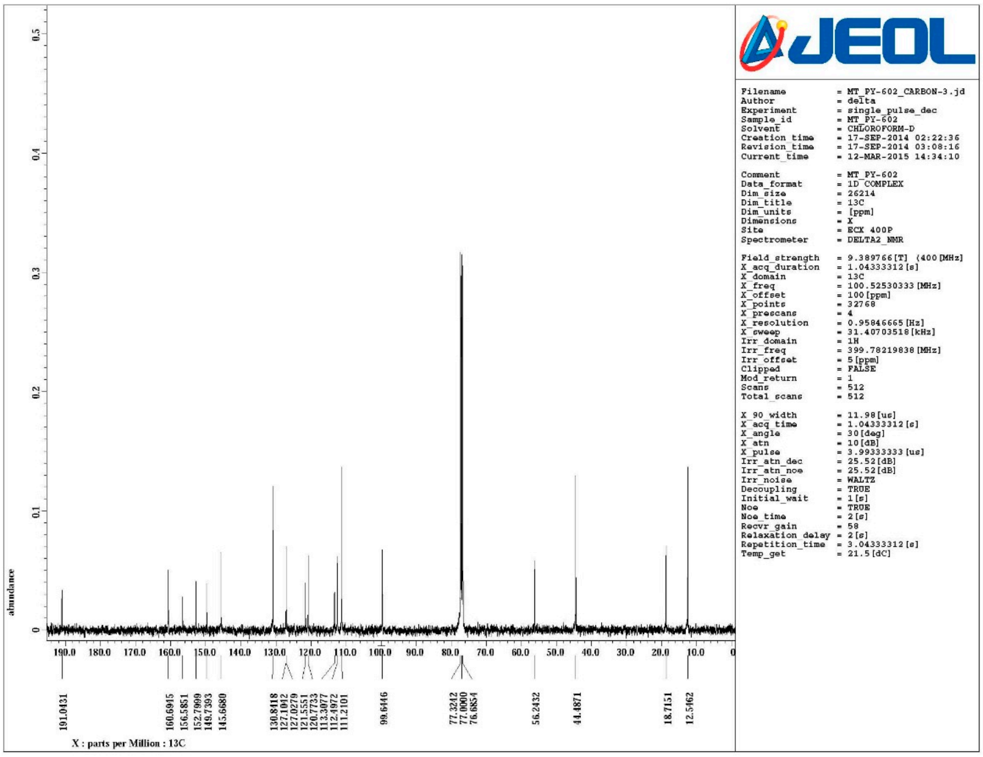


**Figure S10.** 1H-NMR of (E)-6[(3-(4-diethylaminophenyl)acryloyl]-7-methoxy-4-methyl-2H-chromen-2-one (**13**).

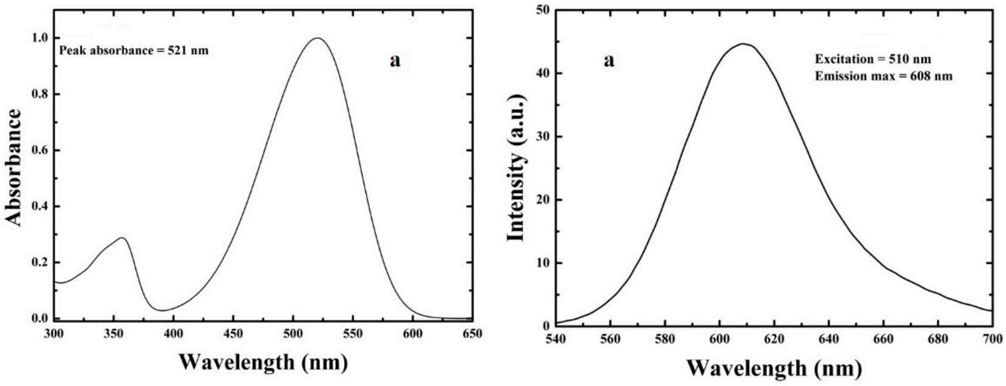


(**a**)

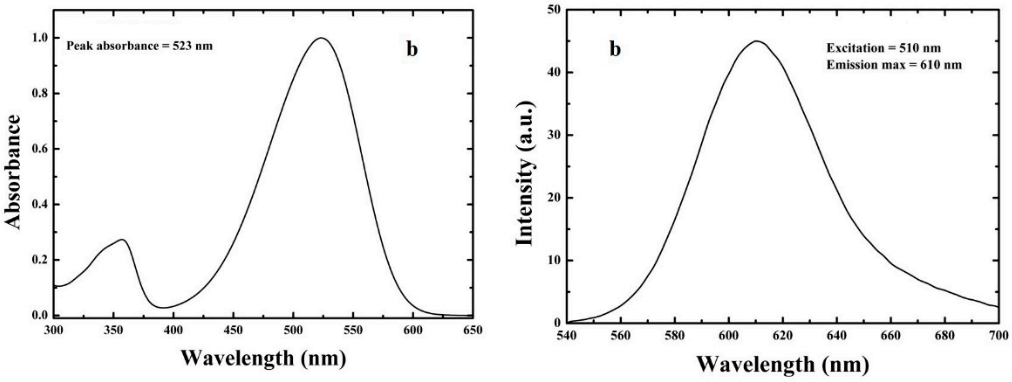


(**b**)

**Figure S11**. Normalized one-photon absorption and fluorescence spectra of compounds **7** (**a**) and **8** (**b**) in DMSO.

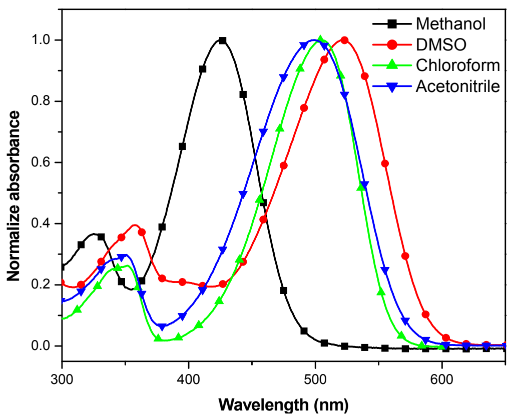


(**a**)

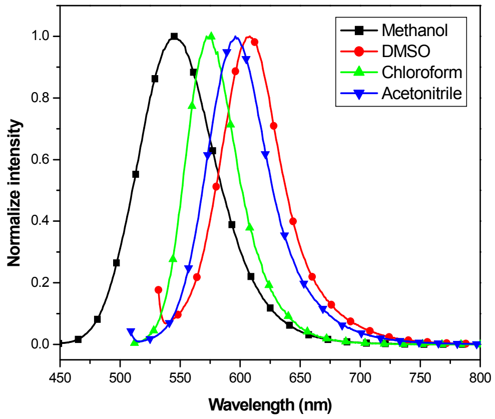


(**b**)

**Figure S12**. Effect of solvent on the absorption and fluorescence spectra of compound **8**.

**Table S1.** Stokes shift in different solvents for compound **8**.

| Solvent | Relative Polarity [1] | Compd. 8 | | |
|---|---|---|---|---|
| | | λab. (nm) | λem. (nm) | Stokes Shift (nm) |
| Methanol | 0.762 | 425 | 546 | 121 |
| Acetonitrile | 0.460 | 499 | 597 | 98 |
| Dimethyl sulfoxide | 0.444 | 522 | 608 | 86 |
| Chloroform | 0.259 | 505 | 572 | 67 |

[1] Christian R. *Solvents and Solvent Effects in Organic Chemistry*; Wiley-VCH Publishers: Weinheim, Germany, 2003.

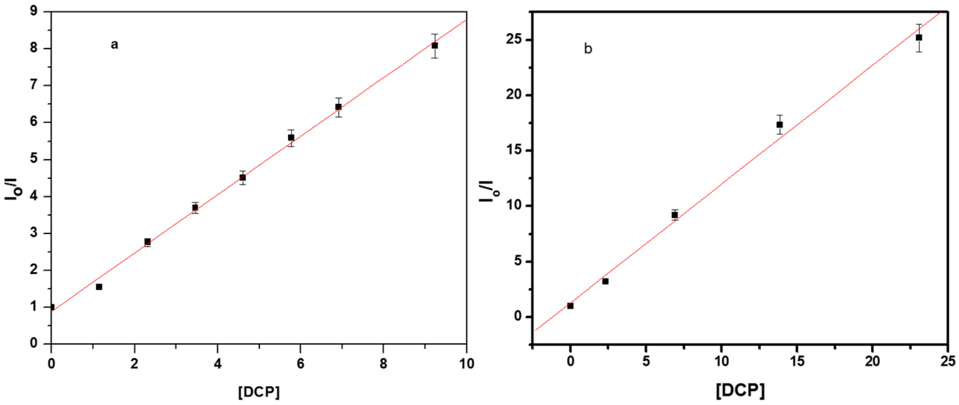


**Figure S13.** Stern-Volmer plot for compounds **7** (**a**) and **8** (**b**) upon DCP addition.

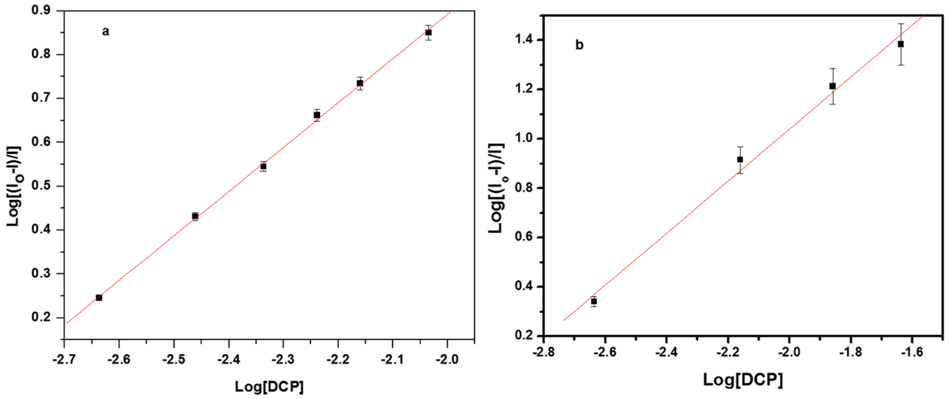


**Figure S14.** Plot for calculation of binding constant (K) and binding sites (n) for compounds **7** (**a**) and **8** (**b**) on increasing concentration of DCP.

**Table S2.** The Stern-Volmer constants of the 6-(4-dialkylaminocinnamoyl) coumarins with DCP in chloroform.

| S.No. | Structure | Ksv($M^{-1}$) |
| --- | --- | --- |
| 7 | | 791 |
| 8 | | 1070 |
| 11 | | 214 |
| 12 | | 208 |
| 13 | | 253 |

Stern-Volmer constant was calculated from the fluorescence titration data of compounds (0.02 mM) 3 mL in a cuvette.

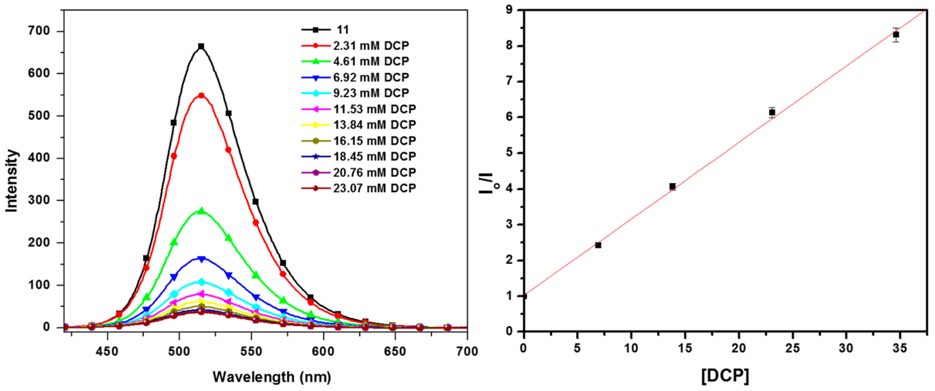


**Figure S15.** Fluorescence spectra for titration of compound **11** with DCP and Stern-Volmer plot.

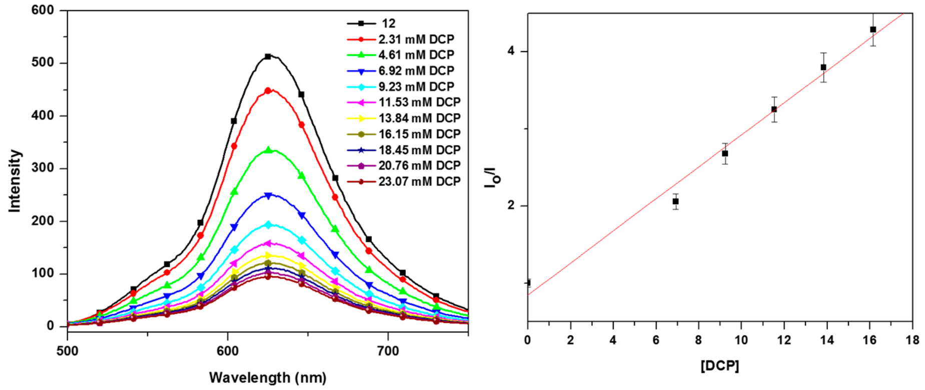


**Figure S16**. Fluorescence spectra for titration of compound **12** with DCP and Stern-Volmer plot.

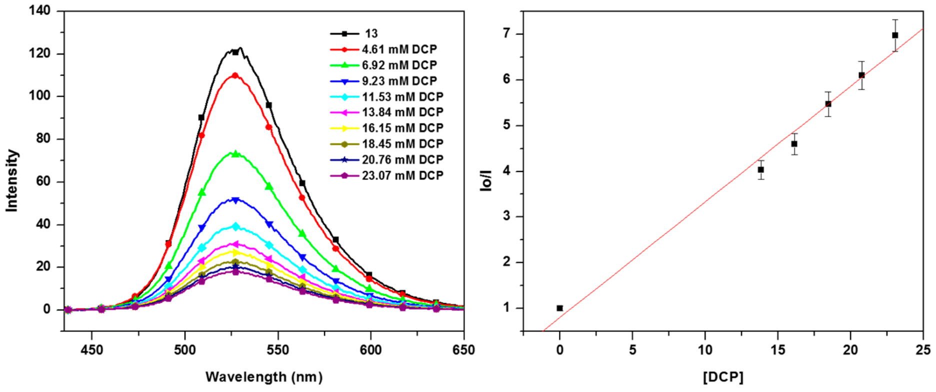


**Figure S17.** Fluorescence spectra for titration of compound **13** with DCP and Stern-Volmer plot.

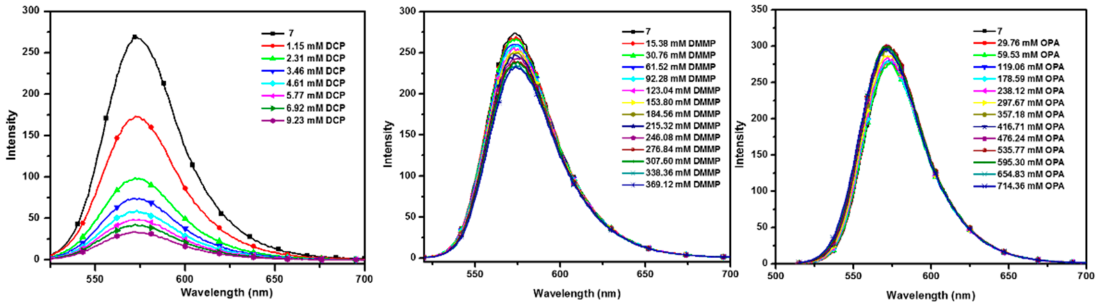


**Figure S18.** Fluorescence spectra for titration of compounds **7** with DCP, DMMP (dimethyl methylphosphonate) and o-phosphoric acid (OPA).

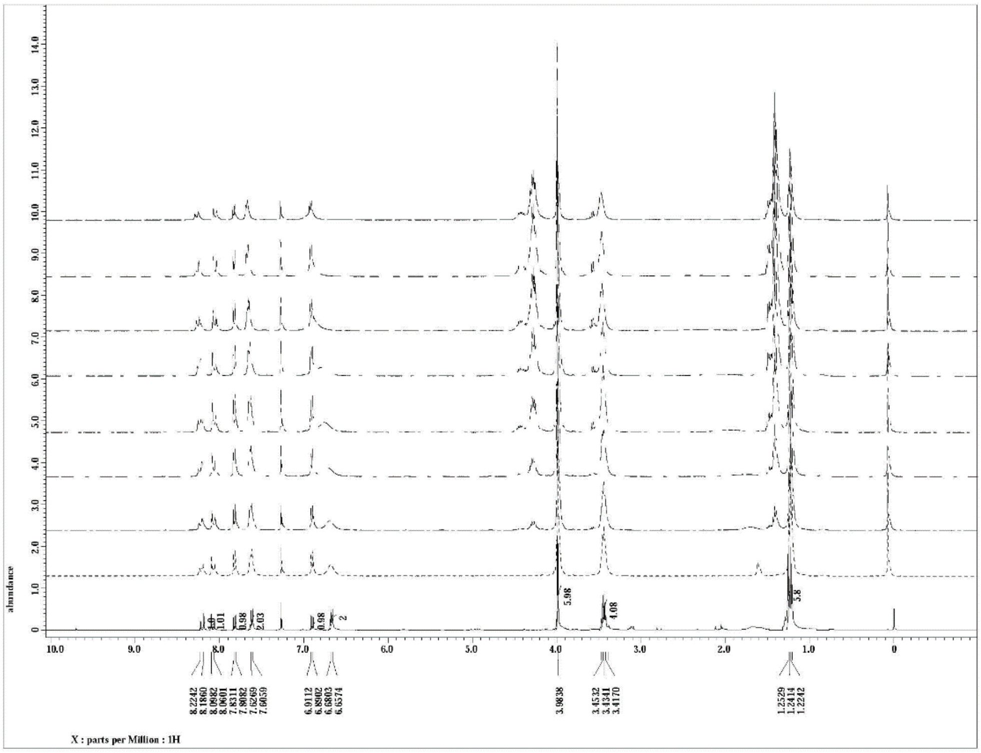


**Figure S19.** 1H-NMR titration plot of compound **8** on addition of DCP (0–4 eq.) in CDCl3.

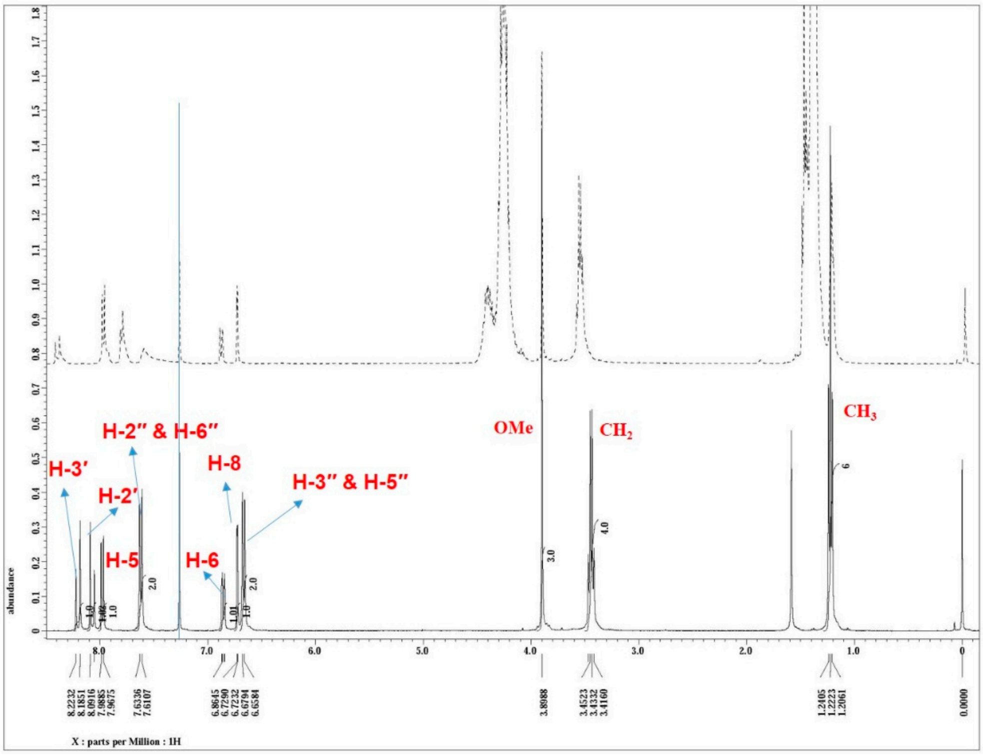


**Figure S20.** 1H-NMR of compound **7** on addition of DCP (4 eq.) in CDCl3.

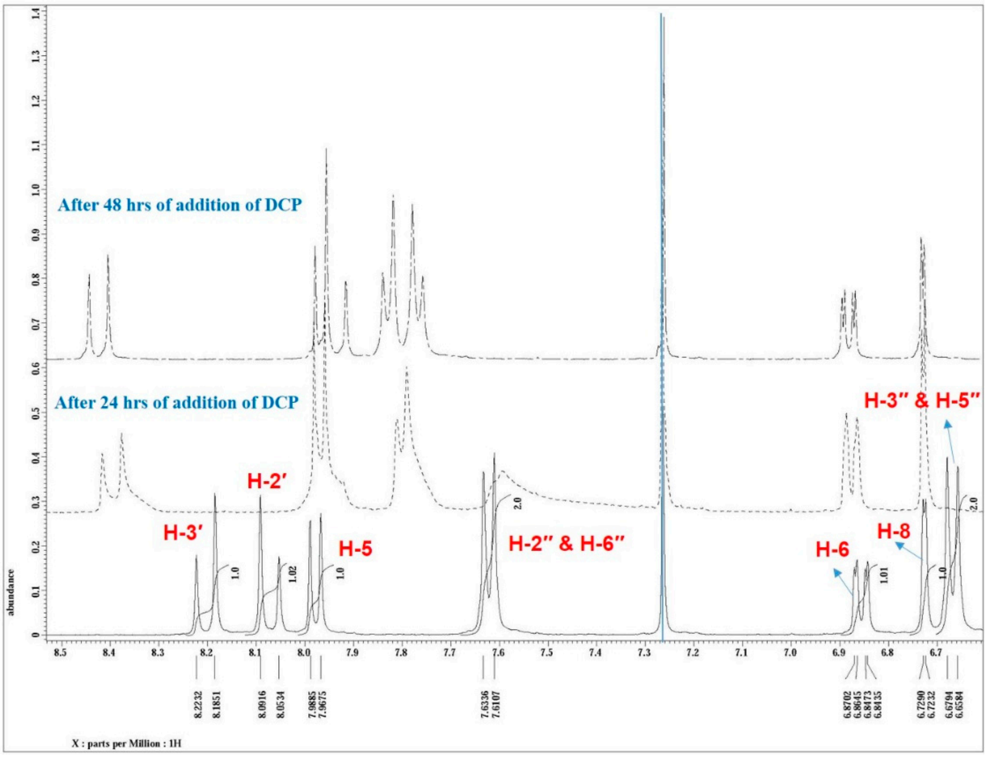


**Figure S21.** 1H-NMR (aromatic region) of compound **7** on addition of DCP (4 eq.) in $CDCl_3$ after 24 and 48 h.
